# Antioxidant Activity and Metabolite Profiling of *Xylocarpus granatum* Extracts Using Gas Chromatography–Mass Spectrometry

**DOI:** 10.3390/metabo13020156

**Published:** 2023-01-20

**Authors:** Rudi Heryanto, Cecep Abdurohman Putra, Munawar Khalil, Mohamad Rafi, Sastia Prama Putri, Alfi Hudatul Karomah, Irmanida Batubara

**Affiliations:** 1 Department of Chemistry, Faculty of Mathematics and Natural Sciences, IPB University, Jalan Tanjung Kampus IPB Dramaga, Bogor 16680, Indonesia; 2 Tropical Biopharmaca Research Center, Institute of Research and Community Services, IPB University, Jalan Taman Kencana No. 3 Kampus IPB Taman Kencana, Bogor 16128, Indonesia; 3 Advanced Research Laboratory, Institute of Research and Community Services, IPB University, Jalan Palem Raya Kampus IPB Dramaga, Bogor 16680, Indonesia; 4 Department of Chemistry, Faculty of Mathematics and Natural Sciences, Universitas Indonesia, Depok 16424, Indonesia; 5 Department of Biotechnology, Graduate School of Engineering, Osaka University, 2-1 Yamadaoka, Suita 565-0871, Osaka, Japan

**Keywords:** antioxidant, GC-MS, metabolite, PCA, *Xylocarpus granatum*

## Abstract

The potential application of *Xylocarpus granatum*, a mangrove species, as traditional medicine has been widely linked to its high secondary metabolite and antioxidant contents. However, few studies have been reported to identify and classify active metabolites responsible for such excellent biological activities. Therefore, the aim of this work was to determine the antioxidant activity, identify the metabolite profiles, and predict the metabolites acting as antioxidants in *X. granatum* extract using a gas chromatography–mass spectrometry (GC-MS)-based metabolomics approach. The seeds, stems, fruit peel, pulp, leaves, and twigs of *X. granatum* were macerated with ethanol. Each extract was analyzed with GC-MS, and the data were processed using mass spectrometry data-independent analysis (MS-DIAL) software to identify the metabolites. The IC_50_ value of plant parts of *X. granatum* ranged from 7.73 to 295 ppm. A total of 153 metabolites were identified and confirmed in the *X. granatum* extracts. Among the identified metabolites, epicatechin and epigallocatechin were the two most abundant in the stem extracts and are expected to have the greatest potential as antioxidants. Principal component analysis (PCA) succeeded in grouping all parts of the plant into three groups based on the composition of the metabolites: group 1 (stems, fruit peel, and twigs), group 2 (seeds and pulp), and group 3 (leaves).

## 1. Introduction

Mangrove is a type of plant that grows in coastal areas and is spread across Indonesia. Extracts and raw materials from mangrove have been widely utilized by coastal people for traditional medicines [1]. One such mangrove species widely used as material for traditional medicine is *X. granatum*. This species is a sea mangrove from the Meliaceae family with a majority of plants spread across Southeast Asia and along the Indian Ocean. In Indonesia, this plant can be found in the Kalimantan and Sulawesi regions [2]. Coastal people still use parts of *X. granatum* plants as traditional medicine, owing to their wide range of biological activities.

Parts of *X. granatum* such as leaves, stems, twigs, and fruit have been reported to contain several secondary metabolites such as alkaloids, flavonoids, monoterpenes, triterpenoids, tetratriterpenoids, limonoids, proanthocyanidins, and phenolic acids [3,4]. These compounds have the potential to exert anticancer, antihyperglycemic, antidyslipidemic, antidepressant, and neuroprotective activities [5,6,7]. Additionally, the extracts of the seeds, stems, fruit peel, and leaves of *X. granatum* can be used as antioxidants and antidiabetics, owing to their polyphenol contents [2,8,9]. The antioxidant activities of *X. granatum* extracts make this species a potential source of one of raw materials for cosmetic skin lightening, which work by inhibiting tyrosinase activity [10]. The biological activities of *X. granatum*, e.g., antioxidant activity, are greatly influenced by its contents of active compounds, which is a crucial factor affecting the quality of *X. granatum* as an antioxidant [2]. 

One method to identify the contents of active compounds in *X. granatum* extracts is by analyzing the metabolite profiles. Metabolite profiling is one method associated with metabolomic approaches that can be used to comprehensively identify primary or secondary metabolites in plants, both qualitatively and quantitatively, and is generally associated with specific metabolite pathways [11,12]. Metabolite profiling facilitates the efficient activity determination and use of active compounds utilization and can be used as a plant quality control process [13].

Comprehensive metabolite profile identification from a complex sample requires a high-resolution analysis method, such as liquid chromatography—mass spectrometry (LC-MS), gas chromatography—mass spectrometry (GC-MS), liquid chromatography—mass spectrometry—mass spectrometry (LC-MS/MS), or capillary electrophoresis—mass spectrometry (CE-MS) [14]. GC-MS analysis is often used for metabolite profiling, owing to its high sensitivity and high resolution, in addition to providing good reproducibility. Another advantage of the GC-MS technique is that it is easy to use and relatively inexpensive in terms of operational cost [15].

In this study, the metabolites in several parts of *X. granatum* plants, i.e., leaves, stems, twigs, fruit peel, pulp, and seeds, were identified using GC-MS. The resulting data were processed with the assistance of MS-DIAL version 4.20 software to identify metabolites; additionally, the compounds acting as antioxidants were predicted based on a comparison of the obtained profile to that of known antioxidant compounds reported in the literature. The result of GC-MS data processing include information on *m/z* values, retention time, retention index, area, and the peak intensity of the identified metabolites. The result of data normalization, which performed with MS-DIAL, was evaluated by PCA (principal component analysis) with SIMCA version 13 software (Umetrics, Umea, Sweden). PCA was used to discriminate every part of *X. granatum* based on similarities in metabolite content.

## 2. Materials and Methods

### 2.1. Samples and Instruments

The plant sample used in this study was *X. granatum* (leaves, stems, twigs, fruit peel, pulp, and seeds) from Togean Islands, Central Sulawesi. All parts of the plant were dried in an oven at 40 °C and ground before extraction.

The following instruments and software were used: GC-MS QP2010 Ultra (Shimadzu, Kyoto, Japan), MS-DIAL version 4.24 software, SIMCA version 13 software (Umetrics, Umea, Sweden), Abf Converter, AMDIS, MORPHEUS, and ChemSketch. The following chemicals were used: N-methyl-N-(trimethylsilyl) trifluoroacetamide (MSTFA), pyridine, alkane mixture (C10-C31), ribitol (internal standard), methanol, chloroform, Milli-Q water, methoxyamine HCl, 2,2-diphenyl-1-picrylhydrazyl (DPPH), and 2-(N-morpholino)ethanesulfonic acid (MES) buffer.

### 2.2. Extraction

The extraction method used was maceration with ethanol. Samples from each plant part (1 g) were soaked in 5 mL ethanol for 24 h, then filtered. The filtrate was concentrated using a rotary evaporator. This extract was used for antioxidant activity tests.

### 2.3. Antioxidant Activity

The antioxidant activity of every plant part of *X. granatum* was determined by DPPH method with referent to the method performed by Batubara et al. (2010) [10]. Each extract was diluted in ethanol to final concentrations of 1.67, 3.33, 6.67, 10.00, 13.33, 16.67, 33.33, 66.67, 100.00, 133.33, and 166.67 μg/mL. The sample aliquot, 100 μL 2-(N-morpholino)ethanesulfonic acid (MES) buffer (pH 7.4), and 100 μL DPPH solution (11.8 mg DPPH in 100 mL ethanol) were added to each well of a 96-well plate. The mixtures were incubated for 30 min; then, the absorbances were read at 514 nm. Vitamin C was used as positive controlm and ethanol was used as the blank. Inhibition activity was calculated using the following formula:

Inhibition (%) = [1 − (Asample − Acontrol)/(Ablank − Acontrol)] × 100%

where Asample is the sample absorbance, Acontrol is the vitamin C absorbance as the control, and Ablank is the ethanol absorbance as the blank. The concentration of each sample and a positive control were tested in triplicate.

### 2.4. Sample Preparation for GC-MS Analysis 

Firstr, 1 mL methanol:chloroform:water (5:2:2) and 100 μg/mL ribitol as an internal standard were added to 10 mg of ground herbal material of every part of *X. granatum* (stems, leaves, pulp, fruit peel, twigs, and seeds). The mixture was homogenized by vortexing and incubated in a shaker for 30 min, then centrifuged at 12,298× *g* at 4 °C for 3 min. The supernatant (600 μL) was transferred into a new tube and mixed with 300 μL Milli-Q water, then recentrifuged under the same conditions described above. Subsequently, 400 μL (sample) and 200 μL (QC sample) of the supernatant were transferred into separate new tubes and dried under vacuum using a centrifugal concentrator at room temperature for 2 h, then dried with a freeze dryer overnight (12 h) before derivatization. The QC (quality control) sample was a mixture of all samples. After drying, derivatization was performed by adding 100 μL methoxyamine HCl in pyridine (20 mg/mL), followed by incubation at 30 °C for 90 min, the addition of 50 μL MSTFA, and reincubation at 37 °C for 30 min in order to induce sylilation before being injected into the GC-MS. Sample derivatization was performed to improve the volatility of the compound for GC-MS analysis.

### 2.5. GC-MS Analysis

The GC-MS analysis performed in this study is a standard procedure used to analyze natural products and was conducted at Fukusaki Lab, Osaka University, Japan [16]. GC-MS QP2010 Ultra (Shimadzu, Japan) with ab InertCap 5 MS/NP column was used, with an injection temperature at 230 °C and an injection volume of 1 μL in split mode (25:1 *v/v*). The carrier gas (He) flow was 3.0 mL/minute, with a linear speed of 39 cm/s. The column temperature was held at 80 °C for 2 min, then increased at a rate of 15 °C/min to 330 °C and held for 6 min. The temperature of the transfer line and the ion source were 250 and 200 °C, respectively. The ions were generated by electron ionization (EI) at 1 kV, and the spectrum was recorded in the mass range of 85−500 *m/z*.

Then, 1 μL pyridine was injected into the GC-MS to check the background, and 1 μL of an alkane compound mixture was used to obtain the n-alkane retention time, which was further used for retention index calculation. Afterwards, pyridine was injected to clean the remaining n-alkane compounds, and 1 μL of the sample blank was injected to check for any contamination. Then, the samples or extracts from every part of *X. granatum* were analyzed. Every sixth sample injection was followed by a QC sample, a sample blank, and pyridine injections in order to check for any contamination and clean the column. For every plant part of *X. granatum*, injection was performed in quintuplicate, with 2 blank injections and 10 QC sample injections. 

### 2.6. Data Analysis with MS-DIAL

The resulting data from GC-MS analysis were obtained as .QGD files. Peak conformation, peak filtering, and annotation were processed using MS-DIAL ver. 4.20 (Riken, Kanagawa, Japan). The output data from GC-MS were converted into CDF files, which were subsequently converted into Abf files with Abf Converter, then imported to MS-DIAL software. Data processing steps and compound identification in MS-DIAL included inputting data as a new project, setting some parameters before analysis (data collection, peak detection, deconvolution, identification, alignment, and filtering), and metabolite identification.

Compound identification at the peak was performed by comparing the conformity of the retention time (RT), retention index (RI), and mass spectrum values from the results of analysis with those of known metabolites in the available library from AllPublic-KovatsRI-VS2. MS-DIAL software identified the metabolites according to the predetermined parameters of analysis. The resulting accuracy of compound identification was confirmed by examining the total score of each compound in the compound search.

After identification was completed, data normalization was performed before the data were used in PCA analysis. Data normalization was performed by determining the ID number of a compound used as an internal standard (ribitol); then, the ID number of the standard compound was input in the ID column of the identified compound.

### 2.7. Data Analysis

All determinations were conducted in triplicate, and the data are reported as average values ± standard deviation (SD).

PCA analysis was achieved using SIMCA version 13 software (Umetrics, Umea, Sweden). The data were exported from MS-DIAL as .txt files, which were then converted into Microsoft Excel files. The Microsoft Excel data were then transposed. PCA classification was expected to be able to group every plant part of *X. granatum* extract based on the metabolite content.

## 3. Results

### 3.1. X. granatum Antioxidant Activity

In this study, the antioxidant activity of every part of *X. granatum* was determined using the DPPH method. As a free radical scavenging method, DPPH, has been widely used to evaluate the antioxidant activity of plant extracts, owing to its rapidity, sensitivity, simplicity, and reproducibility. The results of antioxidant activity are reported as IC_50_, indicating the number of antioxidants needed to lower 50% of the initial concentration of DPPH. Higher IC_50_ values indicate lower antioxidant activity. 

The IC_50_ values of every part of *X. granatum* are presented in Table 1. The antioxidant activities of each part of *X. granatum* vary, with IC_50_ values ranging from 7.73 to 295 ppm. In this study, vitamin C was used as a standard, with an IC_50_ value of 4.18 ppm. Among all plant parts, the stem extract showed the highest antioxidant potential, with an IC_50_ value of 7.73 ppm (Table 1).

### 3.2. X. granatum Metabolite Profiling Using GC-MS

The results of profiling were obtained after confirming the metabolites and performing data curation. One metabolite identified in MS-DIAL was confirmed by conforming the retention index value and the mass spectrum of the compound identified with a known compound found in the AllPublic-KovatsRI-VS2 library. Data curation involves reducing the data on the compounds with the same retention time values and the same compounds with different retention time values. The compound with the highest total score value (750 to 1000) was selected for profiling. The total score shows indicates the similarity of the identified compound relative to a compound in the database.

**Figure 1:**
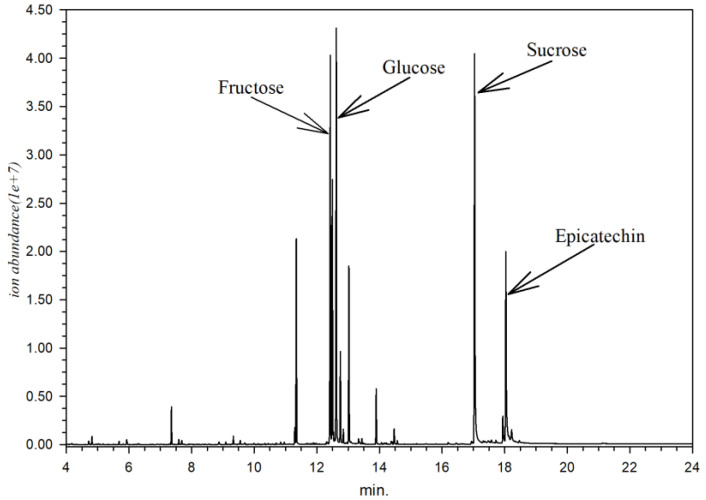
Chromatogram of *X. granatum* stem extract according to GC-MS analysis.

Metabolite identification from every part of *X. granatum* resulted in 153 identified compounds (Table 2). Every part of *X. granatum* is primarily composed of simple sugar (monosaccharides), as reflected by the relative area. The sugar compounds contained in *X. granatum* are sucrose (12.02%), glucose (7.59%), fructose (7.58%), and epicatechin (2.00%), with a chromatogram pattern as shown in Figure 1.

### 3.3. Prediction of Active Antioxidant Compounds in X. granatum

**Figure 2:**
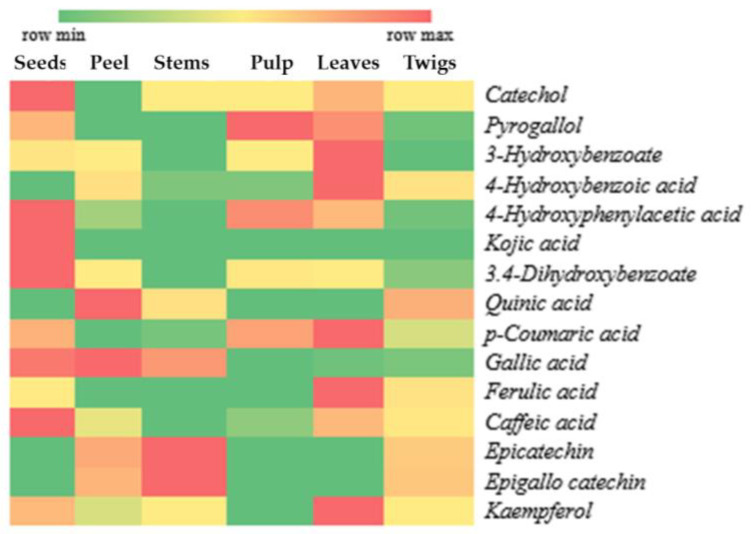
Distribution of antioxidant compounds in plant parts of *X. granatum*.

A total of 15 compounds with potential as antioxidants were identified in the various parts of *X. granatum*, as depicted in the form of a heat map in Figure 2. The heat map is intended to visualize the peak area percentage in a simpler way using the intensity of colors. In Figure 2, the intensity of red color indicates high compound content, whereas the intensity of green color shows indicates low compound content.

According to the results of antioxidant activity testing of *X. granatum* plant parts using the DPPH method, the ethanol extract of *X. granatum* stems has a very high potential compared to the other parts of the plant, as reflected by the lowest IC50 value (Table 1). This result indicates that the antioxidant compounds dominantly found in the stems have higher antioxidant activity compared to those found in the other parts of the plant. Figure 2 shows that the dominant compounds in the stems are epicatechin and epigallocatechin. Besides the stems, these two compounds can be found in the fruit peel and twigs, making the antioxidant activity in the fruit peel and twigs higher than that in the seeds, pulp, and leaves (Table 1).

### 3.4. Discrimination of X. granatum Plant Parts with PCA Based on Metabolite Composition

Discrimination is a process used to differentiate one sample from another by identifying similarities between samples. Samples with many similarities are grouped together; in other words, samples with many differences are separated into differerent groups [17]. The PCA technique is a method of analysis of double variables with the intention of simplifying the observed variables by reducing the dimensions to facilitate the visualization of data grouping and evaluation of similarities among the groups [18]. The application of PCA to the chromatogram enables a reduction in large-sized GC-MS data into several primary components (PCs) so that a two-dimension score plot can show severability among samples. The score plots of the first two components (PC1 and PC2) are usually used in the analysis because these PCs contain the most data variation. 

**Figure 3:**
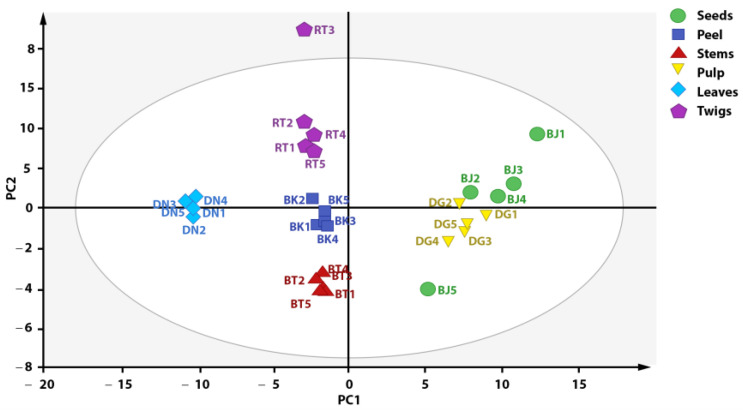
Score plot of PCA analysis before outlier reduction.

The result of PCA analysis is considered acceptable if a small number of primary components can describe a large number of total variations. The result of PCA analysis was visualized as a score plot depicting the grouping of each plant part of *X. granatum* based on the metabolite composition contained in each plant part. Every point in the score plot represents a single sample, and samples with similarities are categorized in the same group [19]. Figure 3 shows the obtained score plot, which explains 91% of total variation (PC1 = 51%, PC = 40%). Samples with the same labels are grouped in adjacent positions. However, some samples did not provide a satisfactory grouping representation, so those data were considered outliers, such as teed 1, seed 5, and twig 3. Therefore, outlier reduction was performed in order to visualize a clearer and better data grouping.

**Figure 4:**
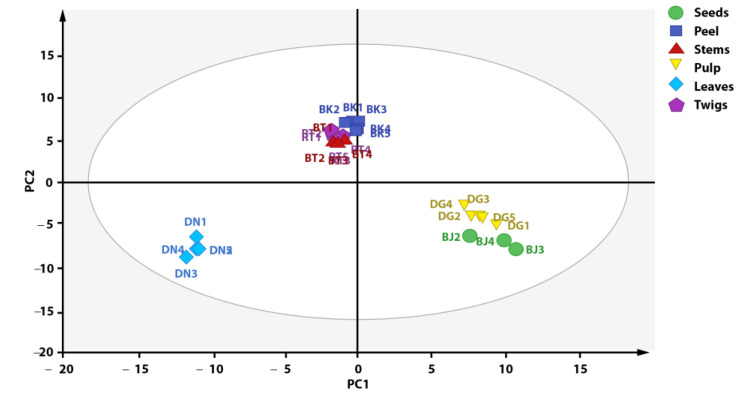
Distribution of antioxidant compounds in plant parts of *X. granatum*.

Outlier reduction resulted in the grouping presented in Figure 4, with an increase in the PC1 value of 1%. Figure 4 shows a plot score that can explain 92% of total variation (PC1 = 52%, PC = 40%). The result of score plot after outlier reduction shows that the metabolite profile of each *X. granatum* plant part can be differentiated into three groups based on metabolite composition, i.e., group 1 (stems, twigs, and fruit peel), group 2 (pulp and seeds), and group 3 (leaves). According to the PCA score plot (Figure 4), the metabolite characteristics of the fruit peel, stem, and twig parts of *X. granatum* are similar, as reflected by the antioxidant activities, with similar IC50 values: 9.02, 7.73, and 9.83 ppm, respectively (see, Table 2). The samples of the pulp and the seeds parts belong to one group, whereas the leaves are separated from the groups containing the other plant parts, showing that there are metabolite composition differences in the leaves. This is also supported by the antioxidant properties of the leaf extract, which has the highest IC50 value (295.08 ppm), showing that it has the lowest antioxidant activity. The association between the results of grouping and the antioxidant activity shows that the compounds with antioxidant activity play significant roles in the grouping of *X. granatum* plant parts.

## 4. Discussion

Metabolite profiling is a technique of analysis used to determine the metabolite profile or the chromatogram pattern of chemical components of an extract with pharmacological activities or chemical components characterizing a plant with the objective of controling the quality [12]. Metabolite profiling and antioxidant compound prediction of *X. granatum* extracts consisted of four stages, i.e., information gathering through GC-MS data analysis using MSDIAL and antioxidant compound prediction through a literature study, data reduction and compilation to convert the spectrum into data that can be processed statistically, PCA multivariate analysis, and review and interpretation of the results of chemometric processes.

An antioxidant is an electron-donor compound that can lower the free radical level and help to reduce or prevent the impact of oxidative stress because of free radicals [20,21]. Common natural antioxidant compounds include vitamin C, vitamin E, carotenoids, phenolic compounds, and polyphenols, which can be grouped as flavonoids, cinnamate acid derivatives, coumarins, tocopherol, and polyfunctional organic acids, respectively [22]. Our literature review of several studies on the properties of antioxidant compounds, as well as the characteristics and applications thereof, indicated that natural metabolite compounds that are usually found in plants commonly include compounds in the group of polyphenols with active groups of hydroxy (-OH) and double-bonded carbons (-C=C-) and function as scavengers and inhibitors of free radicals reactions. Polyphenol secondary metabolites, such flavonoids, polyenes, and compounds containing many -OH groups, can react with free radicals as reducing agents, free radical scavengers, metal-chelating agents, and oxygen-singlet-forming suppressors [23,24,25].

According to our literature review of the properties and the general structures of antioxidant compounds, several compounds contained in *X. granatum* extracts have potential as antioxidants. These compounds mostly belong to the phenolics and polyphenols groups, including catechol [26], pyrogallol [24], 3-hydroxybenzoate, 4-hydroxybenzoic acid, 4-hydroxyphenylacetic acid, 3,4-dihydroxybenzoate [27], kojic acid [28], quinic acid [29], p-coumaric acid, gallic acid, ferulic acid, caffeic acid [30], epicatechin, epigallocatechin, and kaempferol [23].

Simple phenolic compounds such as catechol and pyrogallol are antioxidant compounds with the ability to lower the number of reactive oxygen species (ROS). Pyrogallol is a compound used widely to inhibit oxidation reactions in biodiesel [25]. On the other hand, a catechol-containing polyphenol, epicatechin, is a strong in vitro antioxidant compound, owing to its ability to rapidly lower the level of ROS, bind metallic ions, form inert complexes, and arrest the chain reaction of radical compounds [31].

Another phenolic compound identified as an antioxidant is phenolic acid. Phenolic acid is a strong antioxidant and exhibits antibacterial, antivirus, anticarcinogenic, and anti-inflammatory activities, as well as vasodilatory activity. Phenolic acid may further function as an anticancer agent and prevent heart diseases. Phenolic acid identified in *X. granatum* can be devided into two groups: benzoic acid derivatives and cinnamic acid derivatives. In this study, the identified compounds of benzoic acid derivatives were 3-hydroxybenzoate, 4-hydroxybenzoic acid, 4-hydroxyphenylacetic acid, and 3,4-dihydroxybenzoate, whereas the identified compounds of cinnamic acid derivatives were ferulic acid, gallic acid, p-coumaric acid, and caffeic acid. These compounds were reported to function as effective active antioxidants in radical scavenging of 2-azino-bis(3-ethylbenzthiazoline-6-sulfonic acid) (ABTS), 1,1-diphenyl-2-picryl-hydrazyl (DPPH), and superoxide anions, as well as metal chelation activity in iron ions [27,30,32]. Antioxidant activities of these phenolic acid compounds are influenced by the number of hydroxyl groups (OH-) in the phenyl ring. The length of conjugated double bonds, types of substituents, and the form of the molecules also contribute to the antioxidant activity [20].

In addition to the compounds in the phenolic group, other compounds were identified acting as antioxidants, such as quinic acid and kojic acid. Quinic acid is a carboxylic acid with many hydroxy groups (-OH), with potential as an antioxidant by inhibiting oral pathogens [29]. Kojic acid, on the other hand, is widely used in cosmetic products, especially as a skin-lightening agent [28,33].

## 5. Conclusions

The plant parts of *X. granatum* exhibit varying antioxidant activities. Metabolite profiling in *X. granatum* extracts using GC-MS succeeded in identifying 153 compounds. The compounds expected to have important roles in antioxidant activities are those compounds from the flavonol group, i.e., epicatechin and epigallocatechin, which are dominantly found in the stem of *X. granatum*. Multivariate analysis using PCA succeeded in grouping the plant parts of *X. granatum* into 3 groups based on metabolite composition: group 1 (stems, twigs, and fruit peel), group 2 (seeds and pulp), and group 3 (leaves). The grouping results of *X. granatum* plant parts using PCA can be associated with the antioxidant activities.

## Figures and Tables

**Table 1:** Antioxidant activity of *X. granatum* ethanol extract.

Plant Part	IC_50_ (ppm)
Seeds	104.64 ^e^
Fruit peel	9.02 ^c^
Stems	7.73 ^b^
Pulp	44.48 ^d^
Leaves	295.08 ^f^
Twigs	9.86 ^c^
Vitamin C	4.18 ^a^

The same letter indicates non-significant differences at the 95% confidence interval.

**Table 2:** Metabolites in the extract of *X. granatum*.

No	Rt (minutes)	RI	Ion (*m/z*)	Formula	Compound	Area (%)
1	4.33	1003	117	C_3_H_8_O_2_	propylene glycol	0.028
2	4.60	1028	174	C_3_H_9_N	n-propylamine	0.006
3	4.71	1038	117	C_4_H_10_O_2_	butane-2.3-diol	1.084
4	4.73	1039	152	C_5_H_5_NO	2-hydroxypyridine	0.035
5	4.94	1058	147	C_3_H_8_O_2_	propane-1.3-diol	0.006
6	4.95	1058	130	C_3_H_4_O_3_	Pyruvic acid	0.047
7	5.02	1064	147	C_3_H_6_O_3_	Lactic acid	0.125
8	5.07	1069	131	C_4_H_8_O_3_	2-Hydroxyisobutyric acid	0.341
9	5.18	1078	147	C_2_H_4_O_3_	Glycolic acid	0.356
10	5.39	1097	174	C_4_H_11_N	n-Butylamine	0.003
11	5.55	1111	131	C_4_H_8_O_3_	2-hydroxybutanoic acid	0.003
12	5.87	1141	152	C_5_H_5_NO	4-Hydroxypyridine	0.007
13	5.93	1146	147	C_4_H_8_O_3_	3-hydroxybutyric acid	0.317
14	5.94	1147	116	C_3_H_7_NO_2_	Sarcosine_2TMS	0.007
15	5.95	1148	142	C_4_H_7_NO	butyrolactam	0.036
16	6.68	1216	131	C_4_H_8_O_3_	3-Hydroxyisovaleric acid	0.005
17	6.76	1224	144	C_5_H_11_NO_2_	Valine_2TMS	0.063
18	6.89	1237	189	CH_4_N_2_O	Urea	0.005
19	6.91	1238	116	C_4_H_8_O_3_	4-hydroxybutyric acid	0.011
20	6.95	1242	174	C_2_H_7_NO	Ethanolamine	0.008
21	7.01	1249	175	C_6_H_10_O_4_	ethylsuccinate	0.007
22	7.04	1252	179	C_7_H_6_O_2_	Benzoic acid	0.017
23	7.07	1255	147	C_3_H_6_O_3_	Dihydroxyacetone	0.013
24	7.17	1264	116	C_3_H_7_NO_3_	Serine_2TMS	0.003
25	7.29	1276	174	C_2_H_7_NO	2-Aminoethanol	0.070
26	7.33	1280	158	C_6_H_13_NO_2_	Leucine_2TMS	0.033
27	7.35	1282	147	C_3_H_8_O_3_	Glycerol	0.880
28	7.50	1297	180	C_6_H_5_NO_2_	Nicotinic acid	0.008
29	7.53	1300	158	C_6_H_13_NO_2_	Isoleucine_2TMS	0.033
30	7.69	1317	147	C_4_H_6_O_4_	Succinic acid	0.068
31	7.80	1328	254	C_6_H_6_O_2_	Catechol	0.007
32	7.91	1340	147	C_3_H_6_O_4_	Glyceric acid	0.075
33	7.97	1347	241	C_4_H_4_N_2_O_2_	Uracil	0.010
34	7.99	1349	254	C_4_H_4_O_4_	Fumaric acid	0.027
35	8.14	1365	240	C_5_H_5_NO_2_	pyrrole-2-carboxylic acid	0.013
36	8.21	1372	188	C_3_H_7_NO_2_	Alanine_3TMS	0.020
37	8.25	1376	156	C_6_H_11_NO_2_	DL-Pipecolic acid	0.158
38	8.32	1384	147	C_4_H_6_O_4_	erythronic acid lactone	0.032
39	8.46	1398	218	C_4_H_9_NO_3_	Threonine_3TMS	0.007
40	8.55	1408	239	C_6_H_6_O_2_	hydroquinone	0.021
41	8.55	1409	147	C_5_H_8_O_4_	Glutaric acid	0.069
42	8.61	1415	255	C_5_H_6_N_2_O_2_	Thymine	0.001
43	8.66	1422	103	C_4_H_9_NO_3_	homoserine	0.016
44	8.83	1440	233	C_4_H_8_O_4_	2-deoxytetronic acid	0.004
45	8.86	1443	174	C_4_H_9_NO_2_	3-aminoisobutyric acid	0.080
46	8.96	1456	117	C_10_H_20_O_2_	Decanoic acid	0.004
47	9.17	1480	158	C_5_H_9_NO_3_	trans-4-hydroxy-L-proline	0.012
48	9.23	1487	247	C_5_H_8_O_5_	Citramalic acid	0.003
49	9.33	1498	147	C_4_H_6_O_5_	Malic acid	0.751
50	9.34	1499	223	C_7_H_6_O_2_	p-Hydroxybenzal dehyde	0.011
51	9.43	1509	244	C_4_H_8_N_2_O_3_	asparagine dehydrated	0.004
52	9.45	1512	117	C_4_H_8_O_5_	isothreonic acid	0.022
53	9.48	1516	147	C_4_H_10_O_4_	Threitol	0.031
54	9.53	1521	174	C_4_H_12_N_2_	putrescine 3tms	0.018
55	9.54	1523	267	C_9_H_8_O_4_	Acetylsalicylic acid	0.014
56	9.55	1524	217	C_4_H_10_O_4_	Meso erythritol	0.109
57	9.59	1528	232	C_4_H_7_NO_4_	Aspartic acid_3TMS	0.010
58	9.63	1534	156	C_5_H_7_NO_3_	Pyroglutamic acid_2TMS	0.168
59	9.68	1539	230	C_5_H_9_NO_3_	Hydroxyproline	0.187
60	9.70	1542	174	C_4_H_9_NO_2_	4-Aminobutyric acid	0.455
61	9.81	1556	263	C_6_H_6_O_4_	5-hydroxymethyl-2-furoic acid	0.002
62	9.84	1560	239	C_6_H_6_O_3_	Pyrogallol	0.004
63	9.96	1574	267	C_7_H_6_O_3_	3-Hydroxybenzoate	0.007
64	9.99	1577	147	C_4_H_8_O_5_	Threonic acid	0.097
65	10.01	1580	179	C_8_H_10_O_2_	4-Hydroxyphenethyl alcohol	0.012
66	10.04	1584	129	C_5_H_8_O_5_	2-hydroxyglutaric acid	0.015
67	10.12	1594	147	C_7_H_12_O_5_	2-Isopropylmalic acid	0.124
68	10.28	1614	147	C_6_H_10_O_5_	3-Hydroxy-3-methylglutarate	0.032
69	10.31	1617	217	C_5_H_8_O_5_	xylonolactone	0.009
70	10.35	1622	117	C_4_H_6_O_6_	L-(+)-Tartaric acid	0.034
71	10.41	1630	246	C_5_H_9_NO_4_	Glutamic acid_3TMS	0.007
72	10.46	1636	267	C_7_H_6_O_3_	4-Hydroxybenzoic acid	0.094
73	10.50	1642	200	C_6_H_11_NO_2_	Pipecolic acid	0.037
74	10.55	1648	179	C_8_H_8_O_3_	4-Hydroxyphenylacetic acid	0.004
75	10.74	1672	103	C_5_H_10_O_5_	Xylose	0.013
76	10.82	1683	103	C_5_H_10_O_5_	Lyxose	0.273
77	10.84	1686	271	C_6_H_6_O_4_	Kojic acid	0.285
78	10.98	1703	103	C_5_H_10_O_5_	Ribose	0.095
79	11.14	1726	204	C_6_H_10_O_5_	1.6-Anhydroglucose	0.087
80	11.20	1734	217	C_5_H_12_O_5_	Xylitol	0.016
81	11.22	1736	219	C_6_H_14_O_5_	diglycerol	0.005
82	11.29	1746	117	C_6_H_12_O_5_	Rhamnose	0.252
83	11.30	1746	217	C_5_H_12_O_5_	Arabitol	1.275
84	11.34	1752	117	C_6_H_14_O_5_	6-deoxyglucitol	0.971
85	11.48	1771	147	C_6_H_12_O_5_	2-Deoxy-D-glucose	0.065
86	11.51	1775	297	C_8_H_8_O_4_	vanillic acid	0.020
87	11.52	1777	231	C_6_H_10_O_5_	3.6-anhydro-D-galactose	0.021
88	11.60	1787	156	C_5_H_10_N_2_O_3_	Glutamine_3TMS	0.007
89	11.62	1790	129	C_6_H_14_O_5_	3-deoxyhexitol	0.077
90	11.65	1794	147	C_5_H_10_O_6_	Xylonic acid	0.056
91	11.84	1821	204	C_7_H_10_O_5_	Shikimic acid	0.434
92	11.93	1834	193	C_7_H_6_O_4_	3.4-Dihydroxybenzoate	0.267
93	11.98	1841	204	C_6_H_12_O_5_	1.5-Anhydro-D-glucitol	0.067
94	12.26	1881	159	C_6_H_13_NO_5_	D-(+)-Galactosamine	0.268
95	12.29	1886	103	C_6_H_12_O_6_	Psicose	0.118
96	12.30	1887	103	C_6_H_12_O_6_	Tagatose	0.084
97	12.33	1891	345	C_7_H_12_O_6_	Quinic acid	1.910
98	12.46	1910	104	C_6_H_10_O_6_	L-Gulcono-1.4-lactone	3.503
99	12.50	1916	103	C_6_H_12_O_6_	Fructose	6.983
100	12.53	1921	319	C_6_H_12_O_6_	Mannose	0.179
101	12.56	1925	204	C_7_H_14_O_6_	1-methylgalactose	0.121
102	12.57	1927	319	C_6_H_12_O_6_	Galactose	0.328
103	12.58	1928	217	C_6_H_10_O_6_	Glucono-1.5-lactone	0.998
104	12.62	1935	319	C_6_H_12_O_6_	Glucose	7.042
105	12.71	1947	203	C_6_H_13_NO_5_	Glucosamine	0.023
106	12.74	1953	293	C_9_H_8_O_3_	p-Coumaric acid	0.035
107	12.78	1959	218	C_9_H_11_NO_3_	Tyrosine	0.086
108	12.80	1962	275	C_6_H_12_O_7_	galactonic acid	0.014
109	12.82	1963	204	C_6_H_12_O_6_	hexose	1.079
110	12.84	1968	319	C_6_H_14_O_6_	Mannitol	0.181
111	12.89	1975	281	C_7_H_6_O_5_	Gallic acid	0.034
112	12.90	1977	217	C_6_H_14_O_6_	Galactitol	0.025
113	13.02	1994	318	C_6_H_10_O_5_	conduritol-beta-expoxide	2.131
114	13.10	2006	147	C_6_H_14_O_6_	hexitol	0.099
115	13.31	2040	353	C_5_H_4_N_4_O_2_	Xanthine	0.006
116	13.33	2043	147	C_6_H_12_O_7_	Gluconic acid	0.154
117	13.35	2046	117	C_16_H_32_O_2_	palmitic acid	0.103
118	13.44	2059	204	C_8_H_15_NO_6_	N-Acetyl-D-glucosamine	0.363
119	13.48	2067	147	C_6_H_12_O_6_	myo-inositol	0.019
120	13.53	2073	129	C_8_H_15_NO_6_	N-acetyl-D-mannosamine	0.007
121	13.73	2105	338	C_10_H_10_O_4_	Ferulic acid	0.016
122	13.84	2124	204	C_8_H_15_NO_6_	n-acetyl-d-hexosamine	0.114
123	13.89	2132	217	C_6_H_12_O_6_	Inositol	1.004
124	14.00	2149	219	C_9_H_8_O_4_	Caffeic acid	0.011
125	14.07	2160	319	C_8_H_15_NO_6_	N-Acetyl galactosamine	0.035
126	14.30	2198	204	C_9_H_16_O_9_	beta-mannosylglycerate	0.027
127	14.57	2245	117	C_18_H_36_O_2_	Stearic acid	0.196
128	14.61	2251	202	C_11_H_12_N_2_O_2_	Tryptophan_3TMS	0.011
129	14.69	2266	204	C_9_H_18_O_8_	glycerol-3-galactoside	0.021
130	14.79	2282	361	C_10_H_17_NO_9_S_2_	Sinigrin	0.004
131	15.12	2341	217	C_11_H_15_N_5_O_4_	1-methyladenosine	0.013
132	15.18	2352	204	C_6_H_13_O_9_P	galactose-6-phosphate	0.056
134	15.85	2474	217	C_9_H_12_N_2_O_6_	Uridine_4TMS	0.010
135	16.19	2538	204	C_12_H_22_O_11_	Leucrose	0.130
136	16.89	2674	361	C_12_H_22_O_11_	beta-Lactose	0.015
137	17.03	2702	361	C_12_H_22_O_11_	Sucrose	5.839
138	17.05	2706	204	C_12_H_22_O_11_	D-(+)-Maltose	0.236
139	17.16	2729	217	C_12_H_22_O_11_	D-(+)-Turanose	0.361
140	17.36	2770	217	C_12_H_24_O_11_	lactitol	0.105
141	17.53	2806	217	C_12_H_22_O_12_	lactobionic acid	0.047
142	17.56	2814	361	C_12_H_22_O_11_	Trehalose	0.137
143	17.76	2855	355	C_21_H_22_O_10_	4.5-dihydroxy-7-glucosyloxyflavanone	0.012
144	17.92	2889	361	C_12_H_24_O_11_	Palatinitol	0.009
145	17.94	2893	368	C_15_H_14_O_6_	(−)-Epicatechin	0.422
146	18.08	2925	361	C_12_H_22_O_11_	Gentiobiose	0.033
147	18.14	2937	204	C_12_H_22_O_11_	Melibiose	0.051
148	18.31	2975	456	C_15_H_14_O_7_	(−)-Epigallo catechin	0.032
149	18.74	3072	204	C_12_H_22_O_11_	Galactinol	0.021
150	18.87	3100	219	C_16_H_18_O_9_	Chlorogenic acid	0.008
151	18.93	3109	487	C_15_H_10_O_6_	Kaempferol	0.005
152	20.61	3349	204	C_18_H_32_O_16_	Maltotriose	0.003
153	20.93	3396	361	C_18_H_32_O_16_	Kestose	0.010

## Data Availability

The data presented in this study are available in the main article.
